# Stress enhances the consolidation of extinction memory in a predictive learning task

**DOI:** 10.3389/fnbeh.2013.00108

**Published:** 2013-08-22

**Authors:** Tanja C. Hamacher-Dang, Harald Engler, Manfred Schedlowski, Oliver T. Wolf

**Affiliations:** ^1^Faculty of Psychology, Department of Cognitive Psychology, Institute of Cognitive Neuroscience, Ruhr-University BochumBochum, Germany; ^2^International Graduate School of Neuroscience, Ruhr University BochumBochum, Germany; ^3^Institute of Medical Psychology and Behavioral Immunology, University Hospital Essen, University of Duisburg-EssenEssen, NRW, Germany

**Keywords:** stress, extinction, consolidation, memory, renewal effect, humans, retrieval

## Abstract

Extinction is not always permanent, as indicated by several types of recovery effects, such as the renewal effect, which may occur after a context change and points towards the importance of contextual cues. Strengthening the retrieval of extinction memory is a crucial aim of extinction-based psychotherapeutic treatments of anxiety disorders to prevent relapse. Stress is known to modulate learning and memory, with mostly enhancing effects on memory consolidation. However, whether such a consolidation-enhancing effect of acute stress can also be found for extinction memory has not yet been examined in humans. In this study, we investigated the effect of stress after extinction learning on the retrieval of extinction memory in a predictive learning renewal paradigm. Participants took the part of being the doctor of a fictitious patient and learned to predict whether certain food stimuli were associated with “stomach trouble” in two different restaurants (contexts). On the first day, critical stimuli were associated with stomach trouble in context A (acquisition phase). On the second day, these associations were extinguished in context B. Directly after extinction, participants were either exposed to a stressor (socially evaluated cold pressor test; *n* = 22) or a control condition (*n* = 24). On the third day, we tested retrieval of critical associations in contexts A and B. Participants exposed to stress after extinction exhibited a reduced recovery of responding at test in context B, suggesting that stress may context-dependently enhance the consolidation of extinction memory. Furthermore, the increase in cortisol in response to the stressor was negatively correlated with the recovery of responding in context A. Our findings suggest that in parallel to the known effects of stress on the consolidation of episodic memory, stress also enhances the consolidation of extinction memory, which might be relevant for potential applications in extinction-based psychotherapy.

## Introduction

Just like other types of learning and memory, extinction memory can be subdivided into different phases (Quirk and Mueller, 2008). When extinction is studied in experimental settings, an initial learning phase is usually followed by a phase of extinction learning in which the previously established association between a conditioned stimulus (CS) and an outcome (an unconditioned stimulus (US) or reinforcement) is invalidated by presenting the CS repeatedly without its outcome. After this initial phase of extinction learning, the extinction memory undergoes consolidation. A subsequent encounter of the CS may then trigger retrieval of extinction memory (for a description of this basic paradigm, see for example Myers and Davis, 2002). This retrieval of extinction memory, however, is sometimes prone to failure: Just by the passage of time, the conditioned response (CR) may recover spontaneously (for a review, see Robbins, 1990) and context changes after extinction can cause a renewal of the CR (Bouton and Bolles, 1979; Milad et al., 2005). Thus, extinction does not lead to “forgetting”, but constitutes a new learning process in which a second, inhibitory association between the CS and its outcome is acquired (Rescorla, 1993; Myers and Davis, 2002).

From a clinical perspective, it would often be desirable to strengthen extinction memory and to render it less dependent on the context, as psychotherapeutic treatments of anxiety disorders frequently involve extinction-based methods such as exposure therapy. There have been various attempts to enhance extinction memory via either pharmacological or behavioral manipulations. The approaches which probably received the most attention involve the partial N-methyl-D-aspartate (NMDA) receptor agonist D-cycloserine (DCS), which has been shown to enhance exposure therapy of anxiety disorders (Ressler et al., 2004; Guastella et al., 2008; Norberg et al., 2008), the endocannabinoid system (de Bitencourt et al., 2013; Rabinak et al., 2013) and the reactivation-extinction approach, in which reactivation of the to be extinguished memory is supposed to render this memory labile again, thus making it accessible for permanent extinction (Schiller et al., 2010). In addition, glucocorticoids (GCs) have been suggested as promising enhancers of extinction-based psychotherapy in anxiety disorders (Soravia et al., 2006; Bentz et al., 2010; de Quervain et al., 2011) and posttraumatic stress disorder (PTSD; Suris et al., 2010; Yehuda et al., 2010). It is assumed that this extinction enhancing effect of GCs is mediated via two mechanisms: by impairing retrieval of aversive memories during the exposure session and by enhancing consolidation of extinction memory (Bentz et al., 2010). Enhancing effects of stress and GCs on the consolidation of declarative/episodic memory have often been reported (Cahill et al., 2003; Preuss and Wolf, 2009; Roozendaal and McGaugh, 2011). However, only few studies investigated the impact of stress or GCs on the consolidation of extinction memory. Some evidence from rodent studies suggests that chronic stress might exert impairing effects on extinction and/or extinction retrieval (e.g., Miracle et al., 2006; Garcia et al., 2008; Farrell et al., 2010; Wilber et al., 2011). Most relevant for this study, Akirav et al. (2009) found that acute stress induction after the first extinction trial disrupted extinction on the subsequent day, which might be interpreted as an impairing effect of stress on the consolidation of extinction memory. In contrast, other studies reported accelerated extinction rates under GC administration (Bohus and Lissak, 1968; Barreto et al., 2006) and demonstrated that GC synthesis is a prerequisite for successful consolidation of extinction memory (Barrett and Gonzalez-Lima, 2004; Blundell et al., 2011). Consistently, the GC receptor agonist dexamethasone has been shown to facilitate extinction, whereas the antagonist mifepristone blocked this effect (Yang et al., 2006).

To our knowledge, only one study investigated similar mechanisms in humans (Bentz et al., 2013) by inducing acute stress shortly before extinction learning in a differential fear conditioning paradigm. They found that stress prior to extinction reduced expectancy ratings in men in the retrieval test on the subsequent day (Bentz et al., 2013), which may be indicative of enhanced extinction memory consolidation.

In the present study, we investigated specifically the effects of acute stress on the consolidation of extinction memory by using a predictive learning paradigm (adapted from Ungor and Lachnit, 2006) with acquisition on the first day, extinction followed by stress on the second day and retrieval testing on the third day. We already used this paradigm to investigate effects of stress on extinction memory retrieval in a previous study (Hamacher-Dang et al., 2013), with stress being induced on the third day (prior to retrieval testing). As extinction memory is context-dependent (Bouton, 2004) and contextual cues have been shown to modulate stress effects on memory retrieval (Schwabe and Wolf, 2009), we studied potential modulatory effects of the context by manipulating contextual cues so that initial acquisition of critical associations took place in context A, was extinguished in context B and tested in both contexts (A-B-AB renewal paradigm). We induced stress after extinction by conducting the socially evaluated cold pressor test (SECPT; Schwabe et al., 2008).

Based on the well-known effects of stress or GCs on the consolidation of declarative memory (Abercrombie et al., 2003; Preuss and Wolf, 2009), the enhancing effects of GCs on extinction-based psychotherapy (Soravia et al., 2006; de Quervain et al., 2011) and some of the evidence from the animal studies reported above (Barrett and Gonzalez-Lima, 2004; Akirav et al., 2009; Blundell et al., 2011), we assume that stress after extinction training exerts enhancing effects on the consolidation of extinction memory. This will be reflected by a reduced retrieval of extinguished associations in the retrieval test phase. Due to the context-dependency of extinction memory (Bouton, 2004), the effect will probably be more apparent when memory is tested in the extinction context.

## Materials and Methods

### Participants and procedure

Fifty-seven students were recruited for participation in this study via advertisements and flyers at the Ruhr University Bochum. After exclusion of participants who showed an increase in cortisol concentrations of more than 2.5 nmol/l in response to the control condition (*n* = 4) and exclusion of outliers in the predictive learning task results (*n* = 7, identified via boxplot analysis), the final sample comprised 46 participants (12 men and 10 women in the stress condition, 12 men and 12 women in the control condition). Participants were aged between 18 and 38 years (*M* = 24.7 years, *SD* = 4.2 years) and had a mean body mass index of *M* = 22.9 kg/m^2^, *SD* = 2.2 kg/m^2^. All participants were screened beforehand in a telephone interview; exclusion criteria comprised regular intake of medicine, use of hormonal contraceptives, drug use, smoking, chronic or acute illnesses, and current medical or psychological treatment.

Participants were advised not to consume alcohol or other kinds of drugs within the testing period. In addition, they were told not to consume food and drinks except water and to refrain from physical exercise one hour prior to testing on the second day. They provided written informed consent before the experiment started and received 25€ for their participation at the end of their last test session. The study was approved by the local ethics committee.

Test sessions were conducted in the mornings of three consecutive days (between 9 am and 12 pm). During the first test session (30 min), participants underwent an acquisition phase in a computer-based predictive learning task. In the second test session (60 min) on the following day, the predictive learning task continued with an extinction phase. Directly after extinction, participants were either exposed to stress or to the control procedure. They remained in the laboratory for 25 more minutes to allow for saliva sampling as proof of the stress induction. On the third day, participants were tested for renewal in the predictive learning task (15 min).

### Predictive learning task

In this study, we applied the predictive learning task described in Hamacher-Dang et al. (2013), which is a modified version of the predictive learning task developed by Ungor and Lachnit (2006). In brief, participants were instructed to imagine being the doctor of a fictitious patient who often experiences stomach trouble after having meals in his two favorite restaurants. Each trial started by showing a food stimulus (photos of fruits and vegetables, e.g., apples, carrots) in the center of the screen, surrounded by a colored frame indicating the restaurant (context) in which the food was served. To continue, the participant had to predict whether the patient will suffer from stomach trouble after this meal or not by pressing the corresponding key on the keyboard. After they made their choice, feedback was presented, indicating if the patient actually got sick or not. Feedback was omitted during the renewal test phase on the third day. After an interstimulus interval of 1 sec, the next trial started. Table 1 gives an overview of the task design including the allocation of stimuli to contexts and outcomes. During acquisition and extinction, each of the twelve stimuli of the respective phase was presented ten times. The renewal test comprised four presentations of the four critical stimulus-context combinations. Throughout the predictive learning task, the order of stimulus presentations was randomized block-wise, so that each block contained two presentations of all stimuli of the respective learning phase. Within each block, the presentation order was randomized. Directly before extinction started, one block of reminder trials from the acquisition phase were given.

**Table 1:** **Design of the predictive learning task**.

	**Day 1** **Acquisition phase**	**Day 2** **Extinction phase followed by stressor/control**	**Day 3** **Renewal test phase**
Context A	**a**+, **b**+, o+, c−, d−, p−	k+, l+, s+, m−, n−, t−	**a?, b?, e?, g?**
Context B	**e**+, f+, q+, **g**−, h−, r−	**a**−, **b**−, u−, i+, j+, v+	**a?, b?, e?, g?**
*Trials per stimulus*	10	10	4

Letters indicate the different stimuli; stimuli which were critical for the renewal test are marked in bold. For each participant, fruit and vegetable photos were randomly allocated to the letters. Signs represent the feedback delivered to the participant (+ the patient got stomach trouble, – the patient did not get stomach trouble, ? feedback omitted)

For all analyses, data was averaged over stimuli a and b as they reflected identical contingencies.

### Stressor and control procedure

We applied the SECPT as described in Schwabe et al. (2008). In the stress procedure, participants immersed their right hand into a basin filled with ice-cold water (0–3°C) for 3 min. At the same time, they were being videotaped and monitored by a reserved experimenter. In the control condition, participants immersed their hand into a basin with warm water and were neither being videotaped nor monitored.

#### Blood pressure measures

Blood pressure was measured using Dinamap vital signs monitor (Critikon, Tampa, FL; cuff placed on the upper left arm) before, during and after hand immersion as a marker of sympathetic nervous system (SNS) activity. Due to technical failure, data from one participant could not be obtained and thus had to be excluded from blood pressure analysis.

#### Saliva sampling and analysis

As marker of hypothalamic-pituitary-adrenal (HPA) axis activity, we assessed free salivary cortisol concentrations 1 min before stress induction (baseline) as well as one and 25 min after stress induction. Saliva samples were collected using Salivette sampling devices (Sarstedt, Nümbrecht, Germany) and analyzed with commercial assays (ELISA; IBL International, Hamburg, Germany). Inter and intra assay variations were below 10%. Due to insufficient amounts of saliva, the data of four participants were incomplete and thus had to be excluded from cortisol analysis.

#### Subjective ratings

Directly after the stress or control procedure, participants rated on a scale from 0 (“not at all”) to 100 (“very much”) how stressful, painful and unpleasant they had felt during the procedure (method adopted from Schwabe et al., 2008).

### Statistical analyses

For all statistical tests, the level of significance was set to .05. *P*-values of *t*-tests were corrected for unequal variances where appropriate. Greenhouse-Geisser corrected *p*-values were used if the assumption of sphericity in repeated measures analysis of variance (ANOVA) was violated. Partial correlation analysis controlling for the factor group included the following variables as markers of the stress response: increase in cortisol concentrations from baseline to 25 min after SECPT/control, increase in systolic blood pressure from baseline to during SECPT/control, and the subjective rating of stressfulness. As performance variables, we included the percentage of stomach trouble predictions to the extinguished stimuli a/b+ in the acquisition context and the extinction context.

## Results

### Stress response

The physiological data and the subjective ratings proved that the SECPT successfully induced stress.

#### Salivary cortisol concentrations

In response to the SECPT, the stressed group showed a significant increase in salivary cortisol concentrations on day 2 (see Table 2), as reflected by a significant time × group interaction (*F*(2, 80) = 6.42, *p* < .01, *η*^2^ = .14) in a 3 × 2 ANOVA with the within-subjects factor time (baseline, +1, and +25 after SECPT/control procedure) and the between-subjects factor group (stress vs. control group). In addition, a significant main effect of time emerged (*F*(2, 80) = 5.28, *p* = .02, *η*^2^ = .12). The main effect of group did not reach significance (*p* = .06). T-tests indicated that the stress group had significantly higher cortisol concentrations than the control group when tested 25 min after the SECPT/control condition (*t*(40) = 3.57, *p* < .01), while the two groups did neither differ significantly before (*p* = .27) nor 1 min after SECPT/control (*p* = .83).

**Table 2:** **Salivary cortisol concentrations and blood pressure responses to as well as subjective ratings of the SECPT vs. control procedure**.

	**Control**	**Stress**
*Salivary cortisol (nmol/l)*	
Before procedure	14.99 ± 7.20	18.26 ± 11.73
1 min after procedure	12.42 ± 6.63	12.92 ± 8.29
25 min after procedure	9.67 ± 4.48	18.78 ± 11.43**
*Systolic blood pressure (mmHg)*			
Before procedure	116.28 ± 8.95	116.47 ± 13.13
During procedure	115.81 ± 10.09	132.70 ± 15.27**
After procedure	110.84 ± 7.07	113.27 ± 12.47
*Diastolic blood pressure (mmHg)*			
Before procedure	65.80 ± 5.90	65.77 ± 8.52
During procedure	65.57 ± 5.70	78.55 ± 8.47**
After procedure	64.20 ± 5.89	64.17 ± 8.10
*Subjective ratings after procedure*			
Stressful	5.42 ± 12.85	39.09 ± 17.16**
Painful	0.83 ± 4.08	58.64 ± 18.33**
Unpleasant	6.67 ± 13.08	50.91 ± 20.22**

Stressfulness, painfulness and unpleasantness were rated on a scale from 0 (“not at all”) to 100 (“very much”). Data represents means ± standard deviation. ** *p* < .01, significant difference between stress and control group (*t*-tests).

#### Blood pressure

The SECPT elicited a significant increase in blood pressure in the stress group compared to the control group, as reflected by significant group × time interactions for systolic blood pressure (*F*(2, 86) = 31.04, *p* < .001, *η*^2^ = .42) as well as for diastolic blood pressure (*F*(2, 86) = 41.70, *p* < .001, *η*^2^ = .49). T-tests confirmed that the two groups only differed significantly during hand immersion (see Table 2; *t*(43) = 4.39, *p* < .001 for systolic blood pressure, *t*(43) = 6.06, *p* < .001 for diastolic blood pressure), but neither before nor after hand immersion (all *p* > .41).

#### Subjective ratings

Participants ratings of their subjective feelings during the SECPT or control procedure revealed that stressed participants experienced the situation as significantly more stressful (*t*(44) = 7.58, *p* < .001), painful (*t*(44) = 14.46, *p* < .001) and unpleasant (*t*(44) = 8.73, *p* < .001) than participants of the control group (see Table 2).

### Predictive learning task

#### Acquisition and extinction

**Figure 1:**
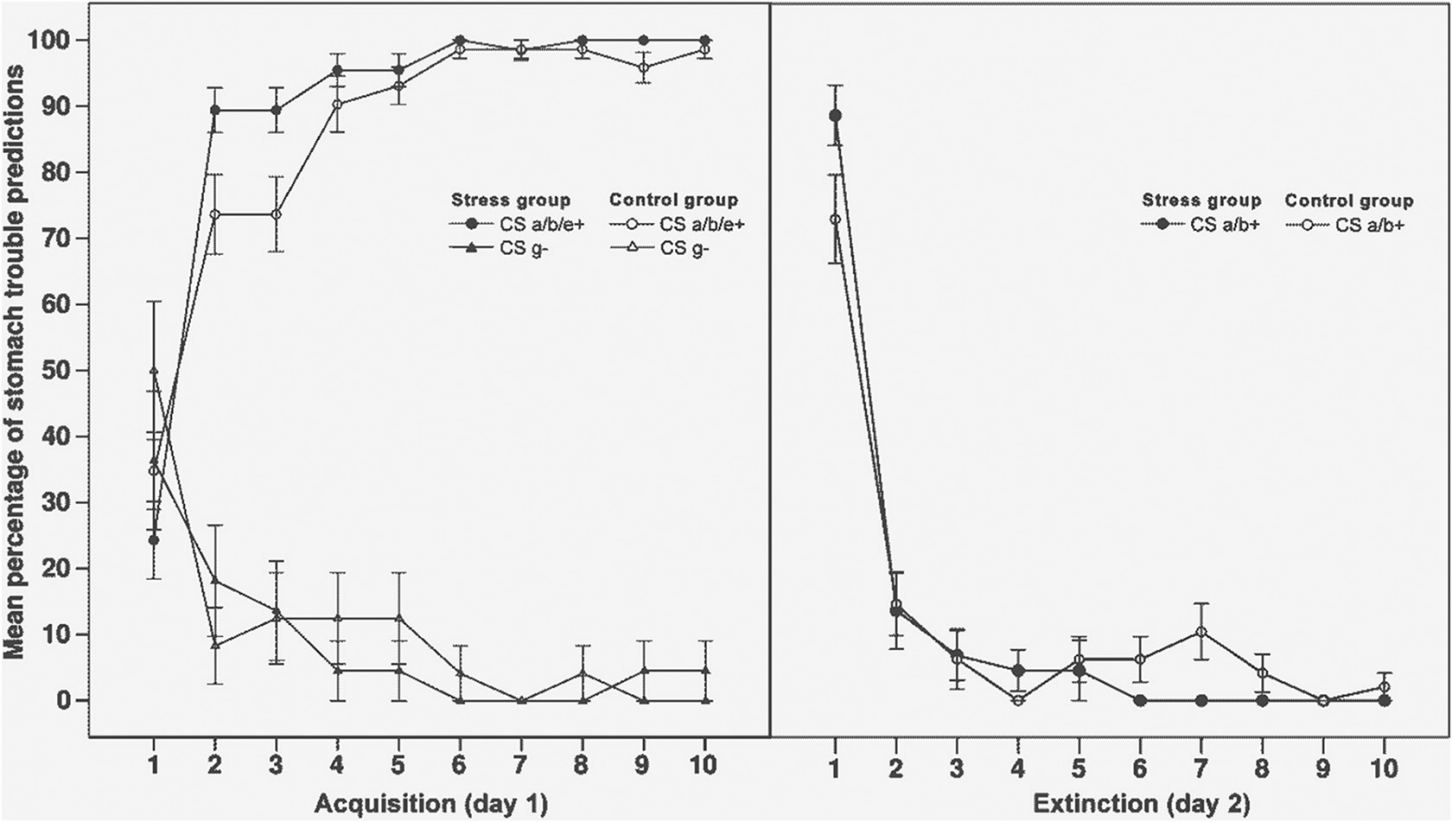
**Mean percentage of stomach trouble predictions to critical stimuli across all trials of the acquisition phase (left side of the graph) and the extinction phase (right side)**. For the acquisition phase, data is averaged over stimuli (CS) a, b and e as they underlay similar contingencies in this phase. CS e+ and g− were not shown during extinction. CS a/b+ were shown in context A during acquisition (day 1) and extinguished in context B (day 2). Error bars indicate standard errors of the mean.

The mean percentage of participants making a stomach trouble prediction to stimulus a/b+ across each trial of the acquisition and extinction phase is shown in Figure 1.

To assess performance in the acquisition and extinction phase, we compared the mean percentage of stomach trouble predictions in the first two trials (beginning) with the mean percentage in the last two trials (end). In the acquisition phase, data was averaged over stimuli a/b+ and e+, as they reflected identical contingencies in this phase. In the extinction phase, we assessed predictions to stimuli a/b+ only, as the control stimuli e+ and g− were not presented during this phase.

For the acquisition phase, a 2 × 2 × 2 ANOVA with the within-subjects factors outcome (stomach trouble associated stimuli a/b/e+ vs. not with stomach trouble associated stimulus g−) and time (beginning vs. end of acquisition) and the between-subjects factor group (stress vs. control) showed a significant main effect of time (*F*(1, 44) = 8.61, *p* < .01, *η*^2^ = .16) and a main effect of outcome (*F*(1, 44) = 331.88, *p* < .001, *η*^2^ = .88), indicating more stomach trouble predictions to stimuli a/b/e+ than to stimulus g−. The significant interaction between time and outcome (*F*(1, 44) = 122.03, *p* < .001, *η*^2^ = .74) reflects an increase in the participants’ ability to distinguish between the stimuli associated with stomach trouble (a/b/e+) and the stimulus which was not associated with stomach trouble (g−) from the beginning to the end of the phase. For the extinction phase, a 2 × 2 ANOVA with the within-subjects factor time (beginning vs. end of the phase) and the between-subjects factor group (stress vs. control) was carried out. The ANOVA also revealed a significant main effect of time (*F*(1, 44) = 237.91, *p* < .001, *η*^2^ = .84), reflecting a decrease in stomach trouble predictions from the beginning to the end of the phase.

For both phases, the main effects of group and the interactions with this factor were not significant (all *p* > .16), indicating that the stress group did not differ from the control group with regard to their performance during acquisition and extinction (i.e., before stress was induced).

#### Results of the renewal test

**Figure 2:**
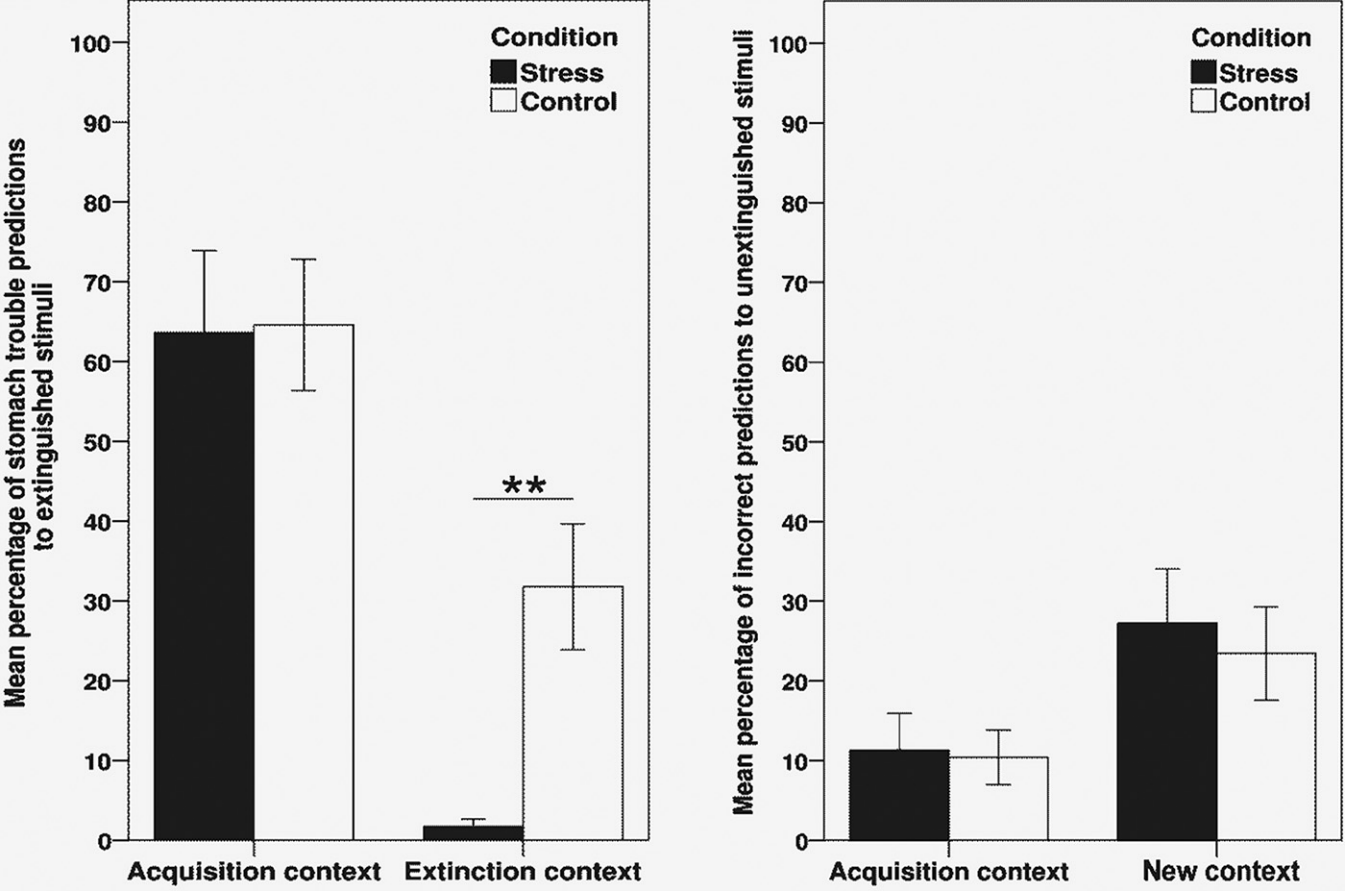
**Results of the retrieval test (day 3), indicating the mean percentage of stomach trouble predictions to the extinguished stimuli a/b+ (left side) and the mean percentage of incorrect predictions to the unextinguished stimuli e+/g− (right side).** Data is shown separately for acquisition context trials and extinction context trials (stimuli a/b+) or new context trials, respectively (stimuli e+/g−), and is averaged over all four trials of the renewal test. ** Significant difference between stress and control group, *p* < .001 (*t*-test). Error bars denote standard errors of the mean.

Figure 2 (left half) displays the mean percentage of participants making a stomach trouble prediction to the extinguished stimuli a/b+, separately for acquisition and extinction context trials. Data is averaged over all four stimulus presentations as there were no significant effects of trial when included as additional within-subjects factor in the subsequent ANOVA. To assess performance in the renewal test phase, we conducted a 2 × 2 ANOVA with the within-subjects factor context (acquisition vs. extinction context) and the between-subjects factor group (stress vs. control). As indicated by a significant main effect of context, participants made more stomach trouble predictions in the acquisition context than in the extinction context, thus reflecting a renewal effect (*F*(1, 44) = 40.27, *p* < .001, *η*^2^ = .48). In addition, the analysis revealed a trend towards a main effect of condition (*F*(1, 44) = 3.76, *p* = .06, *η*^2^ = .08) as well as a trend towards an interaction between context and condition (*F*(1, 44) = 3.80, *p* = .06, *η*^2^ = .08).

Planned comparisons conducted separately for each context indicated that the performance of the stress group did not differ from controls in acquisition context trials (*p* = .94). In extinction context trials, the stress group made significantly less stomach trouble predictions than controls (*t*(44) = 3.78, *p* < .001), thus demonstrating enhanced retrieval of extinction memory.

Regarding the unextinguished control stimuli e+ and g−, stomach trouble predictions in the renewal test were recoded to reflect the percentage of incorrect predictions, which then allowed to average data over the two stimuli. Figure 2 (right half) shows the mean percentage of incorrect predictions to e+/g−, separately for acquisition context trials and new context trials. A 2 × 2 ANOVA with the factors context (acquisition context vs. new context) and group revealed a main effect of context (*F*(1, 44) = 8.47, *p* < .01, *η*^2^ = .16), showing that participants made more mistakes when tested in the “new” context, in which the two stimuli had not been presented before. There was no significant main effect of group (*p* = .67) or interaction with this factor (*p* = .77).

### Correlations

Partial correlation analysis controlling for the factor group showed that the increase in cortisol concentrations from baseline to 25 min after the SECPT was negatively correlated with the percentage of stomach trouble predictions to the extinguished stimuli a/b+ in the acquisition context (*r* = −.38, *p* = .02). Thus, participants with a more pronounced cortisol increase at the beginning of the consolidation phase of extinction memory exhibited a smaller recovery of “stomach trouble” responding when tested in the acquisition context. No significant correlations between the other stress markers and the performance variables emerged (all *p* > .05).

## Discussion

In this study, we investigated the effect of stress on the consolidation of extinction memory in a predictive learning task. As confirmed by salivary cortisol data, blood pressure measures and subjective ratings, stress induction following the extinction session was successful. We found that stress after extinction reduced spontaneous recovery in the extinction context one day later, most probably by enhancing consolidation of extinction memory. Stress induction after extinction did not affect memory for unextinguished associations. Furthermore, we observed a renewal effect, as participants made more stomach trouble predictions to extinguished stimuli in the acquisition context than in the extinction context.

The renewal effect found in our data is consistent with previous studies showing a renewal of conditioned responding after a post-extinction context change, both in fear conditioning paradigms (e.g., Bouton and Bolles, 1979; Milad et al., 2005) and predictive learning (Rosas et al., 2001; Ungor and Lachnit, 2006; Hamacher-Dang et al., 2013).

Taken together with our previous study (Hamacher-Dang et al., 2013), the results indicate that stress exerts opposing effects on extinction memory, depending on the timing of the stressor: while stress prior to retrieval testing impairs the retrieval of extinction memory (see also Deschaux et al., 2013, for an animal study), stress after extinction learning enhances its consolidation and subsequent retrieval. However, in both cases the context might modulate the effect of stress, as for example in this study, the enhancing effect of stress seems to be limited to the extinction context.

An enhancing effect of stress on extinction memory consolidation would also be in line with animal studies showing that GCs are a prerequisite for successful consolidation of extinction memory (Barrett and Gonzalez-Lima, 2004; Blundell et al., 2011; Clay et al., 2011). However, our findings are at variance with those animal studies reporting impairing effects of stress on extinction (Miracle et al., 2006; Garcia et al., 2008; Farrell et al., 2010; Wilber et al., 2011). As most of these studies applied chronic stress prior to conditioning and extinction, diverging findings might be due to differences between acute stress effects and chronic stress with its associated structural changes in the medial prefrontal cortex (mPFC) (atrophy) and Amygdala (hypertrophy) (Roozendaal et al., 2009), likely leading to impaired extinction. In addition, the nonspecific timing of the stressor, probably affecting all phases of the conditioning paradigm, also limits the comparability between these studies. Still, one study also found impairing effects of acute post-extinction stress on subsequent extinction training on the following day (Akirav et al., 2009). This might be due to differences in the experimental paradigms (e.g., aversive vs. non-aversive memories). However, others found beneficial effects of GCs on the consolidation of fear extinction memory (Cai et al., 2006). In sum, the animal literature regarding effects of stress on the consolidation of extinction memory remains somewhat ambiguous and more research characterizing potential mediators and moderators is needed.

Of note, the consolidation-enhancing effect of stress observed in this study parallels results regarding stress or GC effects on the consolidation of declarative memory (e.g., Roozendaal, 2000; Cahill et al., 2003; Preuss and Wolf, 2009). It would also be consistent with the initial finding of reduced US expectancy ratings at test in participants who were exposed to stress before undergoing extinction one day before (Bentz et al., 2013).

The finding that stress after extinction learning reduced the recovery of responding at test only in the extinction context parallels results regarding DCS effects on extinction of fear in animals: DCS has been shown to reduce spontaneous recovery (Vervliet, 2008), when no context change between extinction learning and test occurred, but it did not affect renewal of fear in rats when they were tested in the acquisition context (Woods and Bouton, 2006; Bouton et al., 2008).

In contrast, a very recent study (Haaker et al., 2013) applied the dopamine precursor L-dopa after extinction training and found a reduced renewal effect, suggesting that L-dopa might be a promising agent to strengthen extinction memory independent of contexts. In our study, we found a correlation between cortisol increase and reduced recovery of responding in the acquisition context. This could suggest that the effect of cortisol on the consolidation of extinction memory probably depends on the amount of cortisol secreted in the consolidation phase. A low increase might be sufficient to reduce spontaneous recovery, whereas a somewhat larger cortisol response might be needed to inhibit the renewal effect. Such a dose-dependent effect would be consistent with abundant evidence that in general, effects of GCs on memory consolidation are dose-dependent (Roozendaal, 2000; Abercrombie et al., 2003; Roozendaal, 2003; Andreano and Cahill, 2006; Preuss and Wolf, 2009). In most cases, these studies reported an inverted U-shape relationship between cortisol and enhanced memory consolidation, with moderate levels being the most beneficial. However, we did not find a correlation between cortisol increase and responding in the extinction context. This might be due to the overall low recovery of responding in the stress group, leaving little variance to be explained by variations in cortisol increase.

An alternative explanation for the reduced spontaneous recovery in the stress group might be that SECPT-induced activation of the SNS was sufficient to alter memory consolidation processes in a context-dependent fashion. This view would be supported by studies investigating effects of the noradrenergic drug yohimbine, which has been found to enhance extinction (Cain et al., 2004) while not affecting renewal (Morris and Bouton, 2007). On the basis of our results, it is not possible to further disentangle between GC mediated processes and the role of SNS activity. Current models of stress effects on memory argue that concurrent noradrenergic activation is a prerequisite for the modulatory effects of GCs to occur (Roozendaal and McGaugh, 2011; Schwabe et al., 2012). To further elucidate the mechanisms mediating the enhancing effect of stress on extinction memory consolidation, future studies could apply pharmacological approaches by, e.g., orally administering cortisol or noradrenergic agonists after extinction learning.

As we did not investigate extinction of emotional memories, our results cannot be directly related to studies investigating potential enhancers of extinction-based psychotherapy, which often use fear conditioning paradigms (for reviews, see Kantak and NicDhonnchadha, 2011; Steckler and Risbrough, 2012; de Bitencourt et al., 2013). However, existing parallels between predictive learning and classical conditioning, such as the occurrence of recovery phenomena like spontaneous recovery (Vila and Rosas, 2001b), reinstatement (Vila and Rosas, 2001a) and renewal after a context change (Ungor and Lachnit, 2006; Hamacher-Dang et al., 2013), might warrant some tentative considerations. Our findings support the idea that administering GCs during psychotherapy might be a useful tool to strengthen extinction memory (Soravia et al., 2006; Suris et al., 2010; Yehuda et al., 2010; de Quervain et al., 2011). As our stress manipulation supposedly only affected the consolidation phase of extinction memory, our findings also support the notion that GC administration during psychotherapy acts in part via enhancing consolidation of extinction memory, not only by impairing retrieval of aversive memories (Bentz et al., 2010). Future studies could further investigate these assumed mechanisms of GC action by comparing effects of stress induction before and after extinction learning.

To conclude, the present study showed that stress after extinction in a predictive learning task lead to reduced spontaneous recovery when memory was tested one day later in the extinction context. As stress induction did not affect memory for unextinguished associations, this finding is most likely due to an enhancing effect of stress on the consolidation of extinction memory. When memory was tested in the acquisition context, no differences between the stress and control group emerged. However, the increase in salivary cortisol concentrations after stress induction was negatively correlated with the recovery of extinguished associations in the acquisition context. Thus, sufficient increases in cortisol concentrations might be able to reduce the renewal effect. This would be relevant for clinical applications aiming at reducing relapse after psychotherapeutic treatment. In order to allow more direct conclusions regarding the clinical relevance of our results, future studies should investigate stress effects on the extinction of aversive memories, using fear conditioning paradigms. In addition, a combination of pharmacological studies with a renewal paradigm could help to distinguish between effects of GCs and noradrenergic activation, which might differ in the extent of context dependency.

## Conflict of interest statement

The authors declare that the research was conducted in the absence of any commercial or financial relationships that could be construed as a potential conflict of interest.
